# Supplementary material for: Defects in Galactose Metabolism and Glycoconjugate Biosynthesis in a UDP-Glucose Pyrophosphorylase-Deficient Cell Line Are Reversed by Adding Galactose to the Growth Medium
Source: Int J Mol Sci. 2020 Mar 16;21(6):2028. doi: 10.3390/ijms21062028 (PMC7139386; doi:10.3390/ijms21062028)
Supplement: Supplementary file 1 [file ijms-21-02028-s001.pdf]

Supplementary Table S1: Analysis of protein N-glycosylation in UGP^{-} and UGP^{+} cell using lectin affinity chromatography.

			RCA$^{+,c}$	Complex[b]	Hybrid	Polymannose	[2-^{3}H]GP[a]
			% total [2-^{3}H]GP				*cpm x 10^{-5}*
UGP^{+}	Cells		22.7	48.6	11.3	40.1	5.02
	Medium		23.0	83.3	8.6	8.2	1.48
UGP^{-}	Cells		3.5	35.1	13.1	51.8	5.79
	Medium		5.4	70.2	9.6	20.2	1.65
UGP^{-}	Cells	+ Gal	26.2	35.6	18.0	46.3	3.07
	Medium	+ Gal	37.7	65.6	24.2	10.2	1.93

[a]Cells were rdiolabelled with [2-^{3}H]Man for 18 h as described in Materials and Methods and glycopeptides ([2-^{3}H]GP) were prepared from the cells and media.
[b]Glycopeptides were subjected to ConA-Sepharose chromatography and eluted as described in Materials and Methods to yield fractions containing glycopeptides possessing complex-, hybrid- and polymannose-type N-glycans.
[c]Similarly, glycopeptides were loaded onto RCA-I-agarose columns. After washing the columns, bound glycopeptides (RCA^{+}) were eluted as described in Materials and Methods.
